# Hybrid endoscopic approach for recanalization of biliary-enteric anastomotic occlusion with impacted hepatic stones in Roux-en-Y reconstruction

**DOI:** 10.1055/a-2573-9504

**Published:** 2025-04-11

**Authors:** Jianbo Ni, Fei Li, Kui Peng, Yuqing Mao, Baiwen Li

**Affiliations:** 1 Department of Gastroenterology and Digestive Endoscopy, Shanghai General Hospital, Shanghai Jiao Tong University School of Medicine, Shanghai, China; 2 Shanghai Key Laboratory of Pancreatic Disease, Shanghai General Hospital, Shanghai Jiao Tong University School of Medicine, Shanghai, China

Management of biliary-enteric anastomotic occlusion with impacted biliary stones in Roux-en-Y reconstruction poses significant challenges. Conventional methods like balloon enteroscopy-assisted ERCP (BE-ERCP) often fails in cases of severe stenosis or complex anatomy 1 2 3 . The case reported here demonstrates the successful use of a novel combination of endoscopic ultrasound (EUS)-guided techniques, cholangioscopy-guided laser lithotripsy, and the novel “soft-tip wire-cutting technique” for recanalization, offering an effective and minimally invasive solution ( Video 1 ).

Hybrid endoscopic approach for recanalization of biliary-enteric anastomotic occlusion with impacted hepatic stones in Roux-en-Y reconstruction.Video 1

An 85-year-old woman with a history of Roux-en-Y hepaticojejunostomy presented with recurrent cholangitis and jaundice. Magnetic resonance cholangiopancreatography revealed significant biliary dilation, multiple impacted stones in the hepatic duct, and severe stenosis at the biliary-enteric anastomosis. The patient was referred for endoscopic intervention after multiple episodes of cholangitis had been unsuccessfully managed with antibiotics.

BE-ERCP attempts failed due to the inability to traverse the stenotic anastomosis. Because of the complexity of the stenosis and impacted stones, a combined endoscopic approach was employed. Under general anesthesia, a linear EUS scope was used to identify the dilated left hepatic duct. A 19G fine-needle aspiration needle (Expect; Boston Scientific Corp., Marlborough, MA, USA) was used for puncture, followed by a contrast cholangiogram confirming biliary obstruction ( Fig. 1 ). To maintain biliary drainage and allow time for subsequent stone treatment, a 8-mm×80-mm fully covered self-expanding metal stent (FCSEMS; Micro-Tech Co., Ltd., Nanjing, China) was deployed via endoscopic ultrasound-guided hepaticogastrostomy. A metal clip was used to anchor the stent to the opposing gastric wall, preventing migration ( Fig. 2 ).

**Fig. 1:**
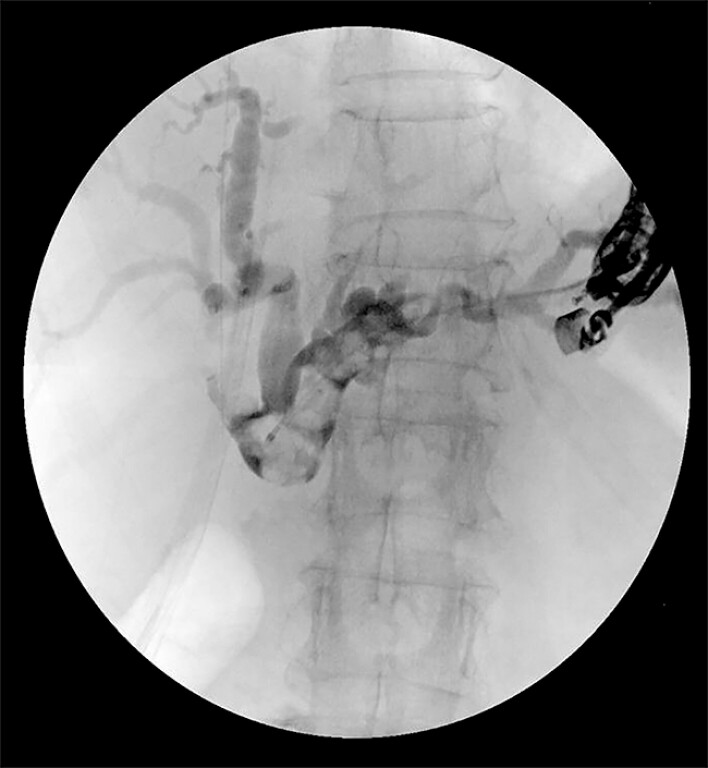
Endoscopic ultrasound-guided hepaticogastrostomy and antegrade cholangiography.

**Fig. 2:**
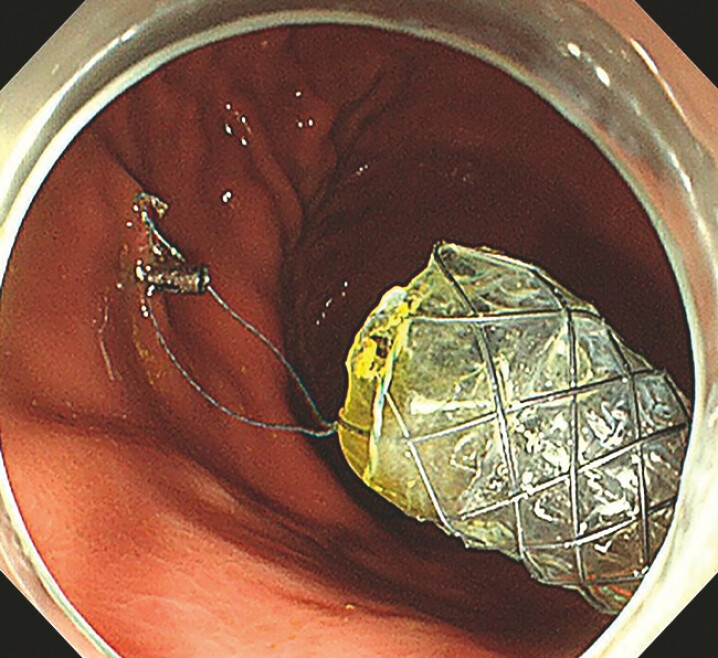
The placement of a fully covered self-expandable metallic stent (FCSEMS) across the endoscopic ultrasound-guided hepaticogastrostomy route was followed by fixation of an endoclip on the opposing gastric wall to prevent stent migration.

After 24 hours, a peroral cholangioscopy (eyeMAX; Micro-Tech) was advanced through the EUS-created tract to visualize the impacted stones. Laser lithotripsy (U100plus lithotripsy laser system; World of Medicine, Berlin, Germany) was performed, fragmenting the stones into smaller, extractable pieces ( Fig. 3 ). The fragmented stones were then removed using a balloon catheter. However, guidewire manipulation through the tight biliary-enteric anastomosis proved challenging. Given the tightness of the biliary-enteric anastomosis and the inability of standard guidewires to traverse the stricture even under direct visualization with the cholangioscope, a novel “soft-tip wire-cutting technique” was applied. In brief, the distal soft tip of the guidewire was cut shorter to increase its stiffness, enhancing its crossability while maintaining its atraumatic properties. With the assistance of the cholangioscope, the guidewire was carefully aligned parallel to the 2bile duct, facilitating the successful traversal of the stricture. Once across the stricture, the guidewire supported a dilating bougie (Cook Medical, Bloomington, IN, USA) and gradual antegrade balloon dilation (6- to 8-mm Hurricane balloon catheter; Boston Scientific). A second FCSEMS (8mm×60mm) was placed at the biliary-enteric anastomosis, ensuring patency and effective treatment of the stricture ( Fig. 4 ).

**Fig. 3:**
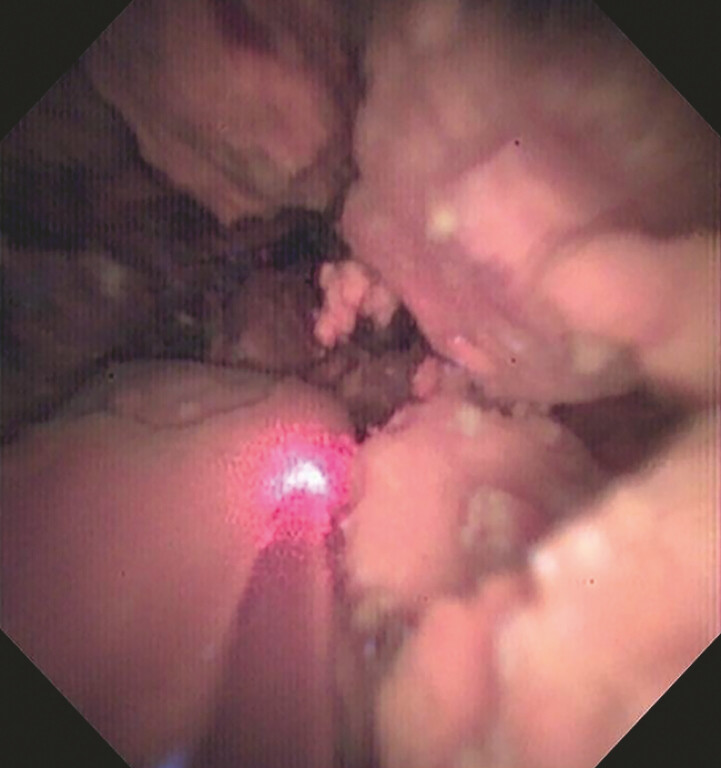
Digital single-operator cholangioscopy-guided laser lithotripsy for crushed impacted stones.

**Fig. 4:**
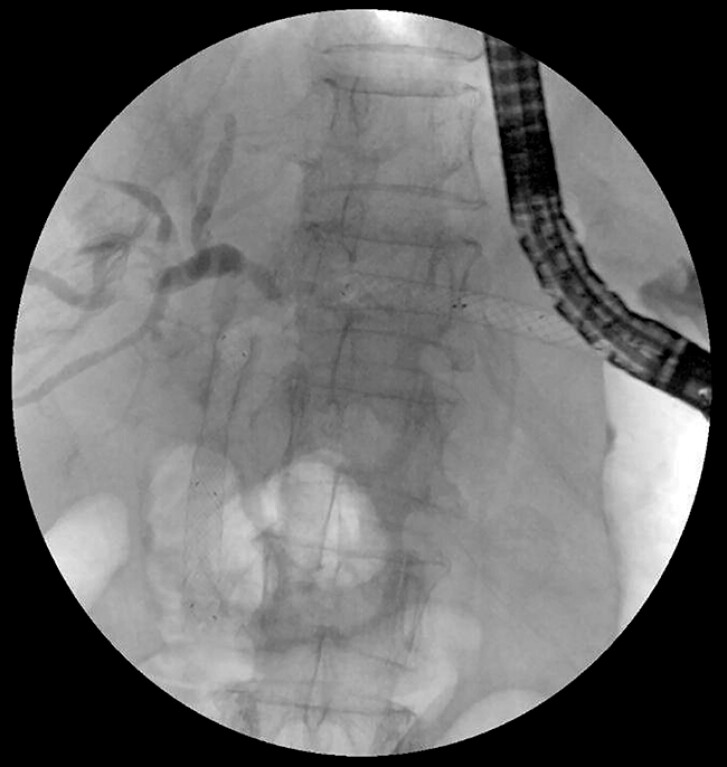
Recanalization of biliary-enteric anastomotic occlusion and placement of a FCSEMS at the biliary-enteric anastomosis.

The procedure was successfully completed without complications. The stents were safely removed after 1 month, when both fluoroscopy and cholangioscopy confirmed sustained patency of the biliary-enteric anastomosis ( Fig. 5 ). The patient remained symptom-free at a 6-month follow-up, with no recurrence of cholangitis or biliary obstruction.

**Fig. 5:**
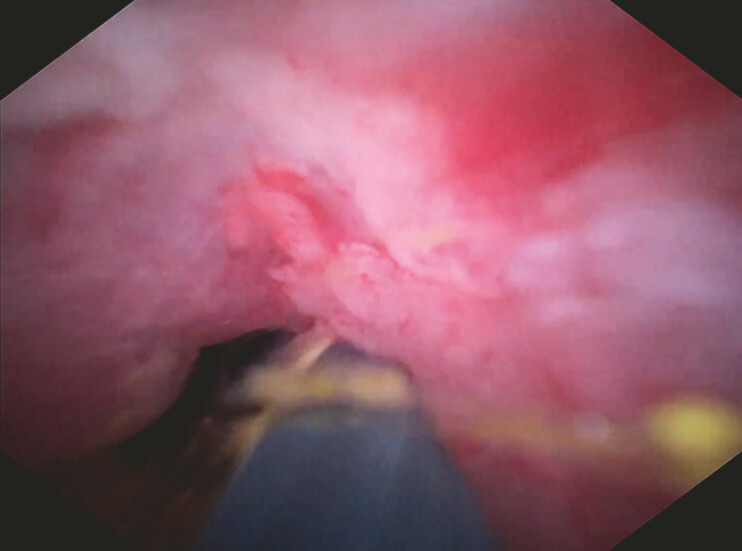
Cholangioscopy confirmed sustained patency of the biliary-enteric anastomosis.

This case highlights the efficacy of advanced endoscopic techniques in managing biliary-enteric anastomotic occlusion and impacted stones in Roux-en-Y reconstruction. EUS and cholangioscopy enable precise access and stone clearance 2 3 4 5 , while the soft-tip wire-cutting technique facilitates traversing tight, fibrotic strictures, offering a minimally invasive and cost-effective solution. The integration of these advanced techniques shows promise for effectively managing complex biliary conditions after surgical alterations.

Endoscopy_UCTN_Code_TTT_1AS_2AD

## Notes

Endoscopy E-Videos https://eref.thieme.de/e-videos
*E-Videos* is an open access online section of the journal *Endoscopy* , reporting on interesting cases and new techniques in gastroenterological endoscopy. All papers include a high-quality video and are published with a Creative Commons CC-BY license. Endoscopy E-Videos qualify for HINARI discounts and waivers and eligibility is automatically checked during the submission process. We grant 100% waivers to articles whose corresponding authors are based in Group A countries and 50% waivers to those who are based in Group B countries as classified by Research4Life (see: https://www.research4life.org/access/eligibility/ ). This section has its own submission website at https://mc.manuscriptcentral.com/e-videos .
